# A genome‐wide association study suggests that *MAPK14* is associated with diabetic foot ulcers†

**DOI:** 10.1111/bjd.15787

**Published:** 2017-11-27

**Authors:** W. Meng, A. Veluchamy, H.L. Hébert, A. Campbell, H.M. Colhoun, C.N.A. Palmer

**Affiliations:** ^1^ Division of Population Health Sciences Ninewells Hospital and School of Medicine University of Dundee Dundee DD2 4BF U.K.; ^2^ Institute of Genetics and Molecular Medicine Western General Hospital, Crewe Road University of Edinburgh Edinburgh U.K.; ^3^ Centre for Pharmacogenetics and Pharmacogenomics Ninewells Hospital and School of Medicine University of Dundee Dundee DD2 4BF U.K.

## Abstract

**Background:**

Diabetic foot ulcers (DFUs) are a devastating complication of diabetes.

**Objectives:**

To identify genetic contributors to the development of DFUs in the presence of peripheral neuropathy in a Scottish cohort with diabetes using a genome‐wide association study.

**Methods:**

A genome‐wide association approach was applied. A case was defined as a person with diabetes (type 1 or type 2) who had ever had a foot ulcer (current or previous) in at least one foot, as well as a positive monofilament test result (i.e. evidence of peripheral neuropathy) recorded in their longitudinal e‐health records. A control was defined as an individual with diabetes (type 1 or type 2) who has never been recorded as having a foot ulcer in either foot but who had a positive monofilament test result recorded in either foot in their longitudinal e‐health records.

**Results:**

There were 699 DFU cases and 2695 controls in the Genetics of Diabetes Audit and Research in Tayside Scotland (GoDARTS) dataset. The single‐nucleotide polymorphism rs80028505 (Chr6p21·31) in *MAPK14* reached genome‐wide significance with a lowest *P*‐value of 2·45 × 10^−8^. The narrow‐sense heritability of this phenotype is 0·06.

**Conclusions:**

We suggest that *MAPK14* is associated with DFUs.

## Abstract 2

**What's already known about this topic?**

- Around 25% of people with diabetes will develop foot ulceration in their lifetime.
- Diabetic foot ulcers (DFUs) account for 85% of all lower‐limb amputations in the U.K.
- There are multiple risk factors for DFUs, the strongest initiating factor being peripheral sensory neuropathy.
- The genetics of DFUs are poorly understood.

**What does this study add?**

- This genome‐wide association study suggests that a skin‐related gene, *MAPK14*, is associated with DFUs in individuals with evidence of neuropathy.
- The narrow‐sense heritability of this disorder, in individuals with evidence of neuropathy, is 0·06.

**What is the translational message?**

- Genetic variants in *MAPK14* are strongly linked with DFUs.
- Further study is needed to confirm the gene's role, followed by clinical trials that should bring significant benefits to patients with DFUs.

Linked Comment: Lainer. *Br J Dermatol* 2017; **177**:1482–1483.

Plain language summary available online

Respond to this article

Diabetic foot ulcers (DFUs) are a major and devastating complication of diabetes. According to the National Institute for Health and Care Excellence guideline, a DFU is defined as a localized injury to the skin and/or underlying tissue, below the ankle, in a person with diabetes.^1^ It has been reported that around 25% of people with diabetes will develop DFUs at some stage in their lifetime.^2^ Although the majority of DFUs (60%–80%) will heal without intervention or after treatment, 10%–15% of them will remain active and 5%–24% of all patients with DFUs will eventually undergo a lower‐limb amputation.^3^ DFUs account for 85% of all lower‐limb amputations and, in the U.K., 50% of bed occupancy in patients with diabetes is because of diabetes‐related foot problems.^4^, ^5^ DFUs have a significant impact on the quality of life of patients, especially those with amputations. These individuals normally have increased disability, high morbidity and higher mortality.^6^ In addition, the cost of treating DFUs is huge. It is estimated that £650 million is spent on foot ulcers or amputations each year by the National Health Service (NHS) in the U.K.^1^ This is equivalent to £1 in every £150 of all NHS costs.

Epidemiological studies have suggested multiple risk factors for DFUs: diabetic neuropathy, peripheral vascular disease, biomechanical factors, previous foot ulceration, poor glycaemic control, longer duration of diabetes, smoking, ethnicity, retinopathy, nephropathy, insulin use, poor vision, age and male sex.^4^ Among these, diabetic neuropathy has been indicated to be the strongest initiating factor for DFUs. A study has shown that 63% of DFUs were as a result of peripheral sensory neuropathy.^7^ This is followed by peripheral vascular disease, which, although not suggested as a cause of ulceration alone, is usually found in combination with diabetic neuropathy and other factors.^8^ Further research on epidemiological risk factors can provide great value in terms of disease prevention and treatment.

At the moment, the role of genetics in DFUs is not clearly understood. It is assumed that DFUs are a common complex disorder determined by both genetic and environmental factors. A previous gene study has suggested that rs699947 in *VEGF* is associated with DFUs.^2^ There is increasing evidence that epigenetic changes (i.e. molecular modification to genes) can have an impact on the development of DFUs by affecting the healing ability of tissues.^9^ So far, there have not been any linkage studies that have reported genetic loci of DFUs. In addition, no genome‐wide association studies (GWASs) have been published so far on DFUs. A GWAS is a hypothesis‐free genetic association study used to identify genes for complex disorders based on phenotype information and genetic information of a population or a cohort.^10^ The purpose of this study was to use a GWAS approach to identify genetic variants for developing DFUs in the presence of peripheral neuropathy, based on phenotype information and genetic information from a Scottish cohort with diabetes.

## Patients and methods

### Participants

The Genetics of Diabetes Audit and Research in Tayside Scotland (GoDARTS) project was established in 2005 to identify genetic risk factors for diabetes and its complications. Participants with and without diabetes are all required to complete a lifestyle questionnaire, a baseline clinical examination and provide biological samples (blood and urine). All participants provided broad informed consent for their health information from the NHS and biological samples to be anonymously linked to the study for future scientific research. The linked health information includes their personal health status, their general practice clinic visits, outpatient appointments, prescribing history and hospital admissions. In addition, participants’ personal information is anonymously linked with the Scottish Care Information‐Diabetes Collaboration (SCI‐DC) database, which is an electronic health (e‐health) record system specifically designed to provide clinical information, to support diabetic screening services and to provide data for national and local audit programmes. Further information about the GoDARTS project and SCI‐DC database can be found in the public domain (http://diabetesgenetics.dundee.ac.uk/ and http://www.sci-diabetes.scot.nhs.uk/). The research followed the tenets of the Declaration of Helsinki. The Tayside Committee on Medical Research Ethics (REC reference 053/04) granted ethical approval for the study. So far, 9439 patients with diabetes have been recruited by the GoDARTS project and 7424 of them have been genotyped using DNA chips. All participants’ health information was anonymously linked with their NHS and SCI‐DC database records from June 1996 until June 2014.

### Definitions of cases and controls

A case of DFU in this study was defined as a person with diabetes [type 1 diabetes (T1D) or type 2 diabetes (T2D)] who had ever been recorded in the linked e‐health records as having a foot ulcer (current or previous) in at least one foot, as well as a positive monofilament test result recorded in the longitudinal e‐health records. A control in this study was defined as a person with diabetes (T1D or T2D) who had never been recorded as having a foot ulcer in either foot in the linked e‐health records but who had a positive monofilament test result recorded in either foot in their longitudinal e‐health records.

The monofilament test is a neurological test for patients with diabetes to check their peripheral sensation.^11^ During the test, a monofilament is pressed at 10 sites on both feet (five sites each) with approximately 10 g pressure for a short time (2 s). Absence of sensation in at least two out of five sites in one foot is a positive test, suggesting peripheral neuropathy.

### Genotyping and quality control

Two sets of DNA chips were applied in the GoDARTS project to genotype participants with diabetes. The Affymetrix SNP6·0 chips (used for 3884 participants; Affymetrix, Santa Clara, CA, U.S.A.) were sponsored by the Wellcome Trust Case Control Consortium 2 (WTCCC2) project and the Illumina OmniExpress chips (used in 3540 participants; Illumina, San Diego, CA, U.S.A.) were funded by the SUrrogate markers for Micro‐ and Macro‐vascular hard endpoints for Innovative diabetes Tools (SUMMIT) project.^12^, ^13^ Both projects (WTCCC2 and SUMMIT) used standard genotyping quality‐control protocols.^12^, ^13^

### Statistical analysis

Software SHAPEIT (https://mathgen.stats.ox.ac.uk/genetics_software/shapeit/shapeit.html) and IMPUTE2 (http://mathgen.stats.ox.ac.uk/impute/impute_v2.html) were applied to impute nondirectly genotyped single‐nucleotide polymorphisms (SNPs) using reference files from the 1000 genome phase I datasets.^14^, ^15^ Badly imputed SNPs were removed based on a cut‐off value (*r* ^2^ < 0·3) suggested by IMPUTE2.

Standard quality‐control steps were frequently applied during data manipulation stages using PLINK (https://www.cog-genomics.org/plink2), such as removal of individuals with > 5% missing genotype data, SNPs with missing genotype of > 5%, SNPs with < 1% minor allele frequency and SNPs that failed Hardy–Weinberg tests (*P* < 0·000001).^16^ SNPs on X, Y chromosomes and mitochondria were not routinely included. The multidimensional scaling (MDS) analysis integrated in PLINK was used to detect population stratification in the cohort. A lambda value (indicating the level of stratification and generated by MDS) should be very close to 1, suggesting minimum ancestry mixture. If two samples in the cohort have a pi‐hat > 0·125 (indicating relatedness), then one of them was removed randomly from further association analysis. Logistic regression tests integrated in PLINK were used to generate association *P*‐values, adjusting for covariates including age, sex, body mass index (BMI), cholesterol, triglycerides, high‐density lipoprotein (HDL), low‐density lipoprotein (LDL), haemoglobin A_1c_ (HbA_1c_) and duration of diabetes. *P*‐values less than 5 × 10^−8^ were considered to be genome‐wide significant variants. The linkage disequilibrium among the top SNPs was also calculated by PLINK.

**Figure 1:**
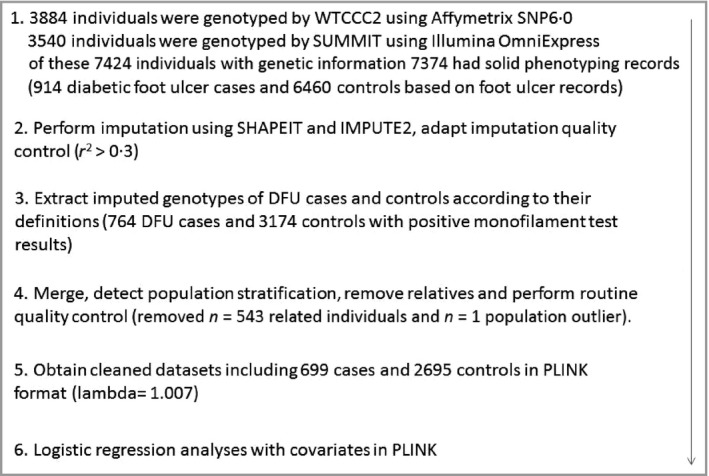
Workflow of the genome‐wide association study on diabetic foot ulcers (DFUs) in Genetics of Diabetes Audit and Research in Tayside Scotland (GoDARTS). WTCCC2, Wellcome Trust Case Control Consortium 2; SUMMIT, SUrrogate markers for Micro‐ and Macro‐vascular hard endpoints for Innovative diabetes Tools.

In this study we also used multiple related GWAS software for different purposes such as SNPnexus (Barts Cancer Institute, Queen Mary University of London, London, U.K.) for SNP functional annotation, HaploView (Broad Institute of MIT and Harvard, Cambridge, MA, U.S.A.) for generating Manhattan plots, LocusZOOM (Department of Biostatistics, Center for Statistical Genetics, University of Michigan, Ann Abor, MI, U.S.A.) for regional visualization and SNPEVG (University of Minnesota, St Paul, MN, U.S.A.) for a corresponding Q–Q plot to evaluate differences between cases and controls caused by potential confounders (e.g. different genotyping laboratories or different DNA extraction methods).^17^, ^18^, ^19^, ^20^ SPSS 22 software (IBM, Armonk, NY, U.S.A.) was used to compare the means of all covariates (except sex) between cases and controls through independent sample *t*‐tests. Sex difference was compared using the χ^2^‐test. The whole workflow is shown in Figure 1. We also calculated narrow‐sense heritability (or chip heritability, estimation of the phenotypic variance explained by the SNPs) based on common SNPs in both chips using GCTA software (http://cnsgenomics.com/software/gcta/#Overview).^21^

## Results

We identified 914 individuals with positive foot ulcer records and 6460 individuals without foot ulcer records from 7424 patients with diabetes with genetic information from the GoDARTS project (50 samples lacked solid phenotyping information). After applying monofilament test results, only 764 DFU cases and 3174 controls with positive monofilament test results were suitable for further analysis based on the definitions used in this study. After removing related individuals (*n* = 543) and population outliers (*n* = 1), we were left with a cleaned study population of 699 DFU cases (463 men, 236 women; 662 with T2D and 37 with T1D) and 2695 diabetic control individuals (1453 men, 1242 women; 2584 with T2D and 111 with T1D).

The prevalence of DFUs in our case–control population was 20·6% [699/(699 + 2695)]. The means of sex, age, BMI, cholesterol, triglycerides, HDL, LDL, HbA_1c_, duration of diabetes were compared between the cases and controls. There were statistically significant differences in sex, LDL, HbA_1c_ and duration of diabetes between cases and controls, whereas there was no significant difference in age, triglycerides, BMI, cholesterol and HDL (Table 1).

**Table 1:** Clinical characteristics of diabetic foot ulcer cases and controls

Covariates	Cases	Controls	*P*‐values
Sex (male : female), *n*	463 : 236	1453 : 1242	**< 0·001**
Age (years)	68·73 ± 9·06	68·48 ± 9·20	0·52
Body mass index (kg m^−2^)	31·22 ± 5·15	31·35 ± 5·41	0·54
Cholesterol (mmol L^−1^)	4·31 ± 0·82	4·37 ± 0·84	0·06
Triglycerides (mmol L^−1^)	2·29 ± 1·33	2·19 ± 1·26	0·09
High‐density lipoprotein (mmol L^−1^)	1·35 ± 0·33	1·36 ± 0·34	0·37
Low‐density lipoprotein (mmol L^−1^)	2·00 ± 0·60	2·07 ± 0·63	**0·01**
Haemoglobin A_1c_ (mmol L^−1^)	7·88 ± 1·50	7·54 ± 1·26	**< 0·001**
Duration of diabetes (years)	21·31 ± 9·00	18·10 ± 8·12	**< 0·001**

Values are mean ± SD, unless otherwise stated. A χ^2^‐test was used to test the difference in sex frequency between cases and controls and an independent *t*‐test was used for other covariates. Results in bold are significant.

**Figure 2:**
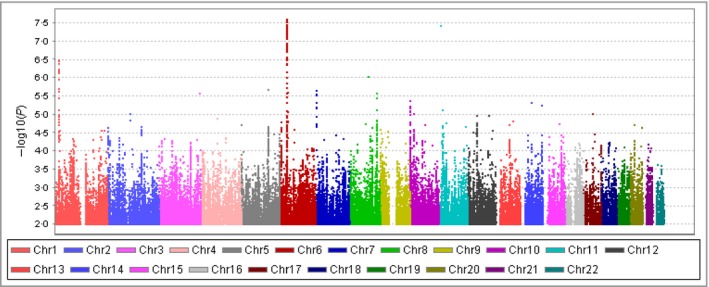
The Manhattan plot of the genome‐wide association study on diabetic foot ulcers (699 cases and 2695 controls). Single‐nucleotide polymorphisms with *P*‐values > 0·01 were not included. Chr, chromosome.

**Figure 3:**
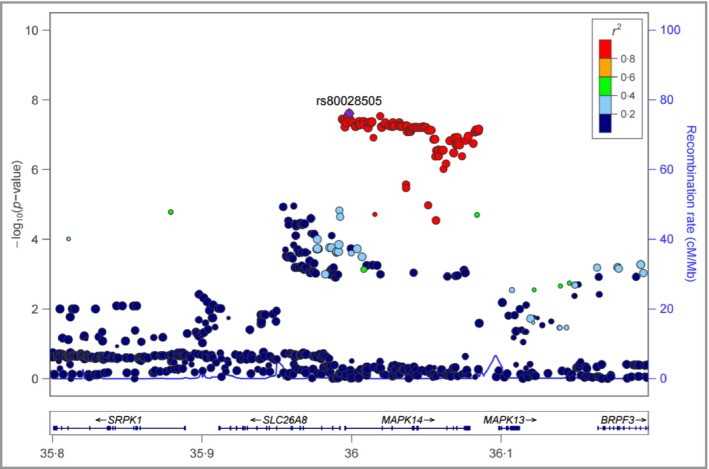
The regional plot of the *MAPK14* region in chromosome 6.

Overall, 6 706 850 genotyped and imputed SNPs were available for association analysis after standard quality‐control steps of genotyping and imputation. No further adjustment based on population stratification was applied since the lambda value was 1·007, indicating a homogeneous population. Logistic regression tests integrated in PLINK were then performed, adjusting for sex, age, BMI, cholesterol, triglycerides, HDL, LDL, HbA_1c_ and duration of diabetes. We identified that the SNP rs80028505 in *MAPK14* reached genome‐wide significance with a lowest *P*‐value of 2·45 × 10^−8^ and an odds ratio of 1·71 (95% confidence interval 1·41–2·06) (Fig. 2, Table 2). A cluster of SNPs in *MAPK14* also showed GWAS significant *P*‐values (*P* < 5 × 10^−8^). The regional plot of the *MAPK14* region is shown in Figure 3. We calculated the linkage disequilibrium among these SNPs (top 10 SNPs with lowest *P*‐values) using our dataset and they are all highly correlated (*r* ^2^ > 0·8) (Fig. S1; see Supporting Information). The Q‐Q plot of the association results is shown in the Figure S2 (see Supporting Information). The narrow‐sense heritability of DFUs (with neuropathy evidence) is 0·06, after adjusting with all covariates. See Table S1 (see Supporting Information) for the results of the GWAS using only individuals with T2D, to remove the influence from the individuals with T1D.

**Table 2:** The top 10 single‐nucleotide polymorphisms (SNPs) of the genome‐wide association study on the diabetic foot ulcers (cases *n* = 699 vs. controls *n* = 2695)

SNP ID	Chromosome position (hg19)	Gene	Minor allele	Minor allele frequency in cases : controls, %	*P*‐value (no adjustment)	*P*‐value	Odds ratio ± standard error	Imputed or genotyped by
rs80028505	6:35998388	*MAPK14*	T	14·02 : 8·99	2·84 × 10^−8^	2·45 × 10^−8^	1·71 ± 0·10	Imputed
rs16883819	6:35997768	*MAPK14*	T	13·97 : 8·97	2·92 × 10^−8^	2·82 × 10^−8^	1·70 ± 0·10	Imputed
rs112201657	6:36019076	*MAPK14*	C	14·25 : 9·20	3·49 × 10^−8^	2·91 × 10^−8^	1·70 ± 0·10	Imputed
rs3761980^a^^,^ b	6:35993906	*MAPK14*	C	13·88 : 8·94	4·01 × 10^−8^	3·57 × 10^−8^	1·69 ± 0·09	Both chips
rs6932598	6:35999080	*MAPK14*	A	13·88 : 8·94	4·23 × 10^−8^	3·76 × 10^−8^	1·69 ± 0·09	Imputed
rs60481532^b^	6:35994942	*MAPK14*	T	13·90 : 8·97	4·43 × 10^−8^	4·01 × 10^−8^	1·69 ± 0·09	Imputed
rs58390233	6:36005100	*MAPK14*	A	13·88 : 8·96	4·72 × 10^−8^	4·20 × 10^−8^	1·69 ± 0·09	Imputed
rs2237096	6:36008002	*MAPK14*	A	13·88: 8·96	4·72 × 10^−8^	4·20 × 10^−8^	1·69 ± 0·09	Imputed
rs56715462	6:36011649	*MAPK14*	G	13·88 : 8·96	4·57 × 10^−8^	4·43 × 10^−8^	1·69 ± 0·09	Imputed
rs61763101	6:35996413	*MAPK14*	T	13·88 : 8·97	4·85 × 10^−8^	4·53 × 10^−8^	1·69 ± 0·09	Imputed

a rs3761980 is also located in the solute carrier family 26 member 8 (*SLC26A8*) gene, which has no evidence relating it to skin.

b rs3761980 and rs60481532 are located in the 5‐upstream region of the *MAPK14* gene whereas other SNPs are located in the intronic regions of the gene.

## Discussion

In Scotland, patients with diabetes are invited to attend an annual free foot screening and to have their feet checked by podiatrists.^22^ The screening aims to identify diabetic foot complications at an early stage to prevent or delay serious consequences such as lower‐limb amputation. During the screening, podiatrists not only record the clinical conditions of foot ulcers, if any (including area, size and depth), but also clinical characteristics that might be linked with DFUs, such as the presence or absence of foot pulses, nerve sensation and vibration functions, previous ulceration history, significant structural foot deformity, presence of callus, amputation history and self‐care ability. However, in the current version (June 2014) of e‐health records provided by SCI‐DC to researchers, the detailed descriptions of ulcers such as area, size and depth are not available. DFUs are categorized as current ulcers (left leg and right leg) and previous ulcers (left leg and right leg) in a longitudinal manner based on examination dates. This is the background to the DFU case definition used in this study.

To achieve a more homogeneous case and control definition, we further adapted positive monofilament test results (evidence of neuropathy) into the sample selection. There were 150 patients with DFUs (16·4% of 914 individuals) who did not have positive monofilament test results but had a positive foot ulcer record (current or previous). This may suggest that the underlying genetic mechanisms of DFUs in these patients might not be the same as for other patients with DFUs (*n* = 764). This stringent definition reduced case numbers and the study power but generated a more homogeneous case population. A similar approach has been successfully applied when defining diabetic neuropathic pain, which is another complication of diabetes (a case should not only have pain evidence provided by prescription records, but also have neuropathy evidence provided by positive monofilament tests).^23^

In terms of controls, 3286 individuals with diabetes but who were foot ulcer‐free (50·9% of 6460 individuals) had negative monofilament results and 3174 individuals had positive monofilament results. Despite the control definition used in the study, we also tried to use the group of 3286 samples as controls, but no SNPs achieved GWAS significance in this study design (Fig. S3; see Supporting Information). This further illustrated the importance of defining a correct homogeneous control population. After removing related samples and population stratification outliers, our current GWAS answered one question: when cases and controls are likely to have diabetic neuropathy, which SNPs (or genetic components) contribute to foot ulceration in a diabetic population?

The prevalence of DFUs (current and/or previous DFUs) in our cohort was 20·6%. This is higher than the generally reported DFU prevalence of 5%–7% in white people.^24^ This is mainly because we adapted monofilament results into the case and control definitions; in particular, we removed a large number of individuals (*n* = 3286) from the controls as a result of lack of evidence of neuropathy. This step is necessary for a genetic study, although it is not normally required to estimate disease prevalence in a general epidemiological study. Furthermore, we also used previous foot ulceration history as part of the case definition to increase the number of cases.

We have identified the SNP rs80028505, which achieved GWAS significance (*P* = 2·45 × 10^−8^, odds ratio 1·71). This SNP was supported by a cluster of nearby SNPs that also showed significant GWAS *P*‐values. The SNP cluster was in *MAPK14*, which is a protein‐coding gene located on chromosome 6. This gene is widely expressed in multiple organs, including skin and soft tissues.^25^ The mitogen‐activated protein kinase (MAPK)14 protein, an enzyme also called p38‐α, is one of the four p38 MAPKs that play an essential role in the cascade of cellular responses evoked by extracellular stimuli such as proinflammatory cytokines or physical stress leading to direct activation of transcription factors.^26^ Evidence from a diabetic mouse model has suggested that p38 MAPK was phosphorylated in wounded skin and using a p38 MAPK inhibitor, the level of phosphorylation was significantly reduced and wound healing was accelerated. This was evidenced by reduced wound width, accelerated re‐epithelialization, increased granulation and reduced inflammatory cell infiltration into the wound.^27^

However, the effect of the MAPK pathway on wound healing is controversial in some studies. For example, activation of the MAPK pathway has been suggested to promote cell collective migration, a biological process involved in tissue formation and repair.^28^ By applying a MAPK inhibitor to a diabetic rat wound model, the rate of wound healing was reported to be reduced by 20%.^29^ It was also reported that MAPK inhibitors can reverse cutaneous wound‐healing effects in a nondiabetic mouse wounding model.^30^ In fact, both acute and chronic wound healing abilities are impaired in diabetes and the MAPK pathway has been confirmed to be activated.^31^, ^32^, ^33^ The MAPK pathway is also involved in other types of ulcers, such as venous ulcer, gastric ulcer and corneal ulcer.^34^, ^35^, ^36^, ^37^ Most SNPs in *MAPK14* affect *MAPK14* expression (*P* = 10^−7^) according to the Genotype‐Tissue Expression (GTEx, Broad Institute of MIT and Harvard, Cambridge, MA, U.S.A.) portal, particularly in skin.^38^

There was a statistical difference in the sexes between cases and controls, indicating sex is a risk factor for DFUs. This is consistent with other studies suggesting that men are more likely to have DFUs.^39^

We have moderate power for this GWAS study. Calculated by CaTS, we have 80% power based on 699 cases and 2695 controls, assuming a minor disease allele frequency of 0·25, a genotypic relative risk of 1·40, a prevalence of DFUs in the diabetic population of 20% and a significance level of 5 × 10^−8^.^40^ The narrow‐sense heritability of this phenotype was 0·06; this heritability excludes effects of gene–gene interactions and gene–environment interactions, for example.

When defining cases and controls in the study, we only considered neuropathy as evidenced by a positive monofilament test, since it is the strongest risk factor.^7^ We did not consider characteristics such as the status of foot pulses, which indicates the existence of peripheral vascular disease. This greatly decreased the complexity of defining samples and statistical analysis. We also included GWAS results using individuals with T2D only and here the *P*‐values of the top SNPs increased slightly. This was probably as a result of the reduced samples size. The reported SNP (rs699947) in *VEGF* was not associated with DFUs in our dataset (*P* = 0·53).^2^

In conclusion, we propose that *MAPK14* is associated with DFUs in a Scottish cohort with diabetes using a GWAS approach. Replication studies and functional studies of this gene will help to confirm its role in DFUs and are needed to provide insights that will facilitate the treatment of DFUs.

## Supporting information


**Fig S1.** The linkage disequilibrium of the top 10 single‐nucleotide polymorphisms with the lowest *P*‐values.

**Fig S2.** Q–Q plot comparing expected and observed –Log_10_(*P*)‐values.

**Fig S3.** The top 10 single‐nucleotide polymorphisms based on a genome‐wide association study design using diabetic foot ulcer cases with positive monofilament test results and diabetic controls with negative monofilament test results.

**Table S1** Top 10 single‐nucleotide polymorphisms of the genome‐wide association study on diabetic foot ulcers in individuals with type 2 diabetes only.

**Video S1** Author video.
