# Supplementary figures and images for: A genome‐wide association study suggests that MAPK14 is associated with diabetic foot ulcers
Source: Br J Dermatol. 2017 Nov 27;177(6):1664–70. doi: 10.1111/bjd.15787 (PMC5829525; doi:10.1111/bjd.15787)

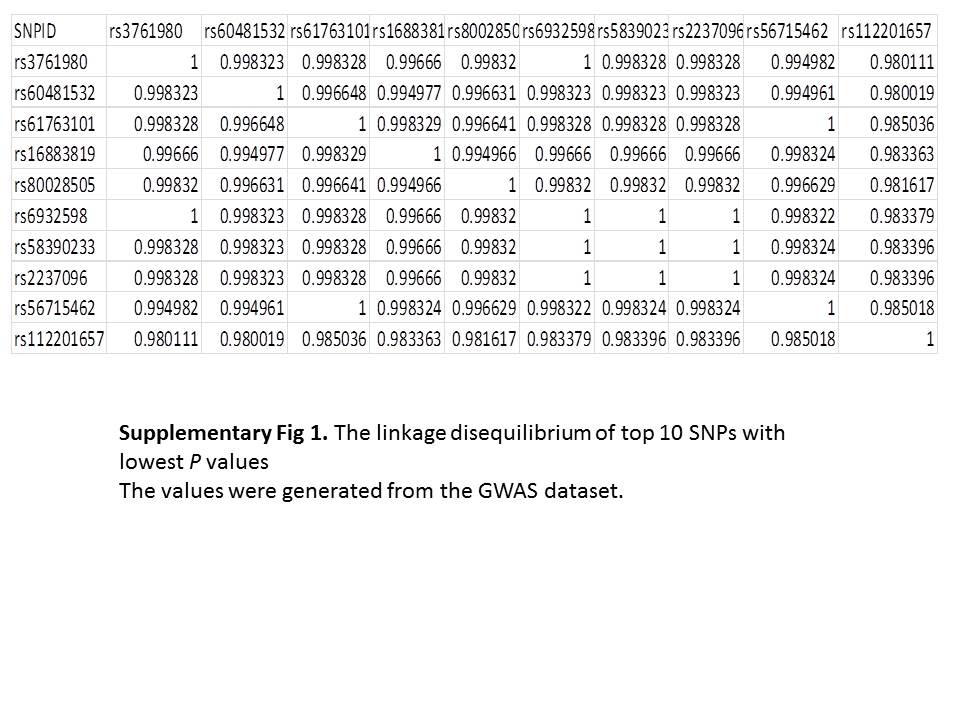

Supplement: Supplementary file 1 — Fig S1. The linkage disequilibrium of the top 10 single‐nucleotide polymorphisms with the lowest P‐values. [file BJD-177-1664-s001.jpg]

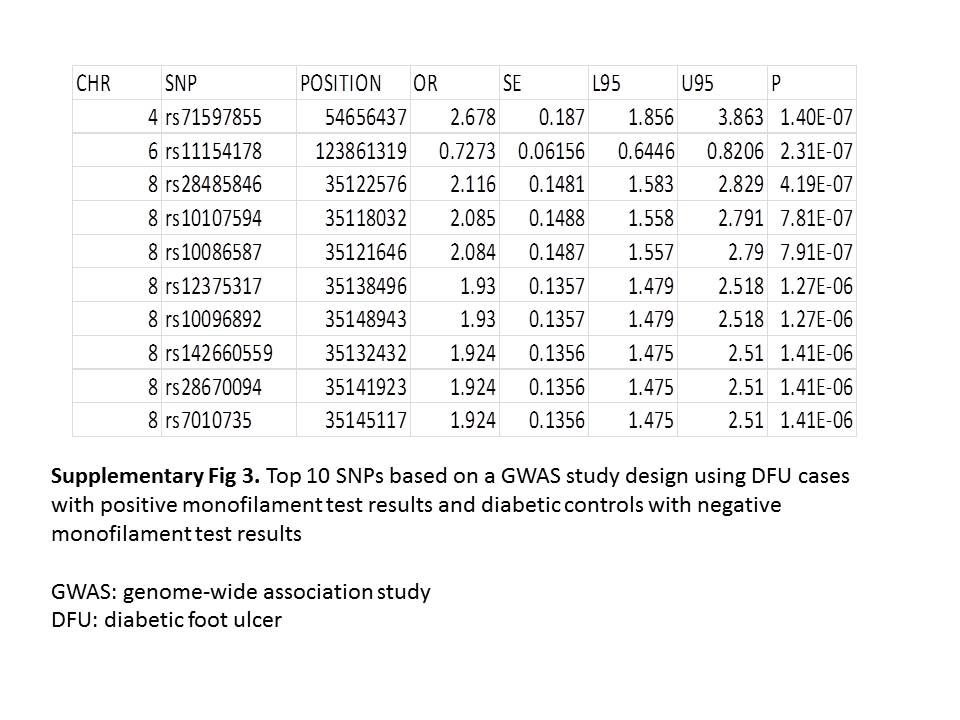

Supplement: Supplementary file 3 — Fig S3. The top 10 single‐nucleotide polymorphisms based on a genome‐wide association study design using diabetic foot ulcer cases with positive monofilament test results and diabetic controls with negative monofilament test results. [file BJD-177-1664-s003.jpg]
