# Supplementary material for: A genome‐wide association study suggests that MAPK14 is associated with diabetic foot ulcers
Source: Br J Dermatol. 2017 Nov 27;177(6):1664–70. doi: 10.1111/bjd.15787 (PMC5829525; doi:10.1111/bjd.15787)
Supplement: Supplementary file 2 — Fig S2. Q–Q plot comparing expected and observed –Log10(P)‐values. [file BJD-177-1664-s002.pptx]

## Slide 1
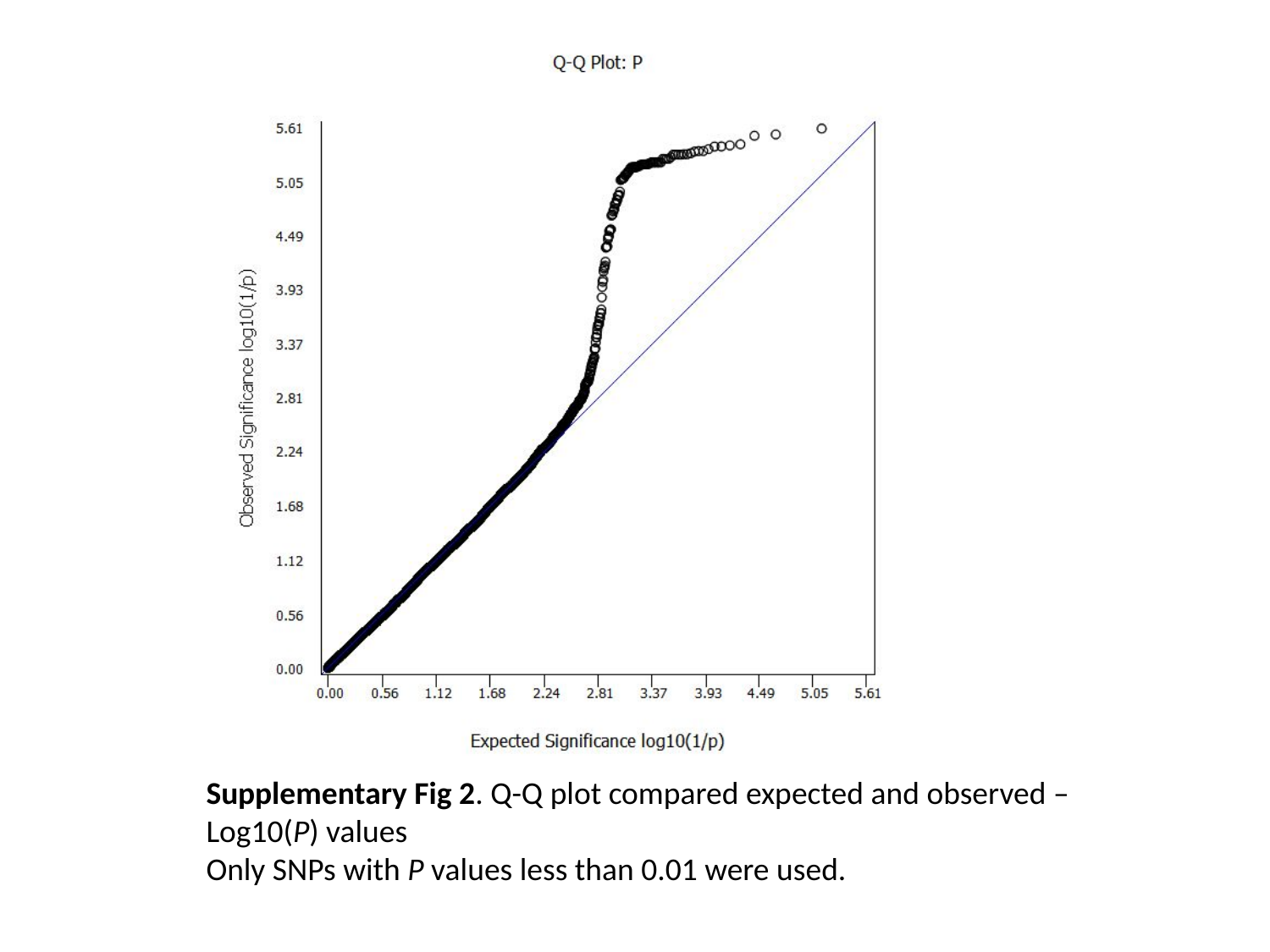

Supplementary Fig 2. Q-Q plot compared expected and observed –Log10(P) values
Only SNPs with P values less than 0.01 were used.
