# Supplementary material for: A genome‐wide association study suggests that MAPK14 is associated with diabetic foot ulcers
Source: Br J Dermatol. 2017 Nov 27;177(6):1664–70. doi: 10.1111/bjd.15787 (PMC5829525; doi:10.1111/bjd.15787)
Supplement: Supplementary file 4 — Table S1 Top 10 single‐nucleotide polymorphisms of the genome‐wide association study on diabetic foot ulcers in individuals with type 2 diabetes only. [file BJD-177-1664-s004.doc]

**Supplementary table 1** Top 10 SNPs of the GWAS on the diabetic foot ulcer on type 2 diabetes samples (cases N=662 VS controls N=2,584)

| SNPID | Chromosome position | Gene | Minor Allele | *P* value |
| --- | --- | --- | --- | --- |
| rs3761980 | 6:35993906 | *MAPK14* | C | 8.89x10^-8^ |
| rs60481532 | 6:35994942 | *MAPK14* | T | 1.15x10^-7^ |
| rs61763101 | 6:35996413 | *MAPK14* | T | 1.14x10^-8^ |
| rs16883819 | 6:35997768 | *MAPK14* | T | 7.16x10^-8^ |
| rs80028505 | 6:35998388 | *MAPK14* | T | 6.10x10^-8^ |
| rs6932598 | 6:35999080 | *MAPK14* | A | 9.33x10^-8^ |
| rs58390233 | 6:36005100 | *MAPK14* | A | 1.04x10^-7^ |
| rs2237096 | 6:36008002 | *MAPK14* | A | 1.04x10^-7^ |
| rs56715462 | 6:36011649 | *MAPK14* | G | 1.11x10^-7^ |
| rs112201657 | 6:36019076 | *MAPK14* | C | 1.07x10^-7^ |

SNP: single nucleotide polymorphism

SNPs were sorted based on their chromosome positions.
